# The Effect of Aerobic Exercise on the Oxidative Capacity of Skeletal Muscle Mitochondria in Mice with Impaired Glucose Tolerance

**DOI:** 10.1155/2022/3780156

**Published:** 2022-06-07

**Authors:** Dan Wang, Dong-Mou Jiang, Rong-Rong Yu, Lin-Lin Zhang, Yan-Zhong Liu, Jia-Xin Chen, Hai-Chun Chen, Yi-Ping Liu

**Affiliations:** ^1^Provincial University Key Laboratory of Sport and Health Science, School of Physical Education and Sport Sciences, Fujian Normal University, Fuzhou, China; ^2^Key Laboratory of Kinesiological Evaluation General Administration of Sport of China, Fujian Province, China

## Abstract

**Methods:**

Male C57BL/6J mice were randomly divided into six different experimental groups (8 animals/group): (1) normal group (NOR), (2) normal control group (NC), (3) normal + exercise group (NE), (4) IGT group (IGT), (5) IGT control group (IC), and (6) IGT+ exercise group (IE).The exercise group received aerobic exercise for 8 weeks. After the intervention, a blood glucose meter was used to detect the level of glucose tolerance in the mouse's abdominal cavity; a biochemical kit was used to detect serum lipid metabolism indicators, malondialdehyde, and superoxide dismutase levels; the ELISA method was used to detect serum insulin and mouse gastrocnemius homogenate LDH, PDH, SDH, and CCO levels. Western blot method was used to detect the protein expression levels of NOX4, PGC-1*α*, and Mfn2 in the gastrocnemius muscle of mice.

**Results:**

(1) Mice with high-fat diet for 30 weeks showed impaired glucose tolerance, insulin resistance, and lipid metabolism disorders. The level of LDH, PDH, SDH, and CCO in the gastrocnemius homogenate of mice was reduced. The expressions of NOX4 protein were significantly upregulated, while the expressions of PGC-1*α* and Mfn2 proteins were significantly downregulated. (2) 8-week aerobic exercise improved the disorders of glucose and lipid metabolism in IGT mice and increased homogenized LDH, PDH, SDH, and CCO levels, and the expressions of NOX4, PGC-1*α*, and Mfn2 proteins in the gastrocnemius muscle of mice were reversed. It is speculated that aerobic exercise can accelerate energy metabolism.

**Conclusion:**

(1) C57BL/6 mice were fed high fat for 30 weeks and successfully constructed a mouse model of reduced diabetes; the mice with reduced diabetes have impaired glucose tolerance, insulin resistance, and lipid metabolism disorders; (2) 8 weeks of aerobic exercise improve glucose tolerance, reduce glucose tolerance in mice, reduce insulin resistance, improve lipid metabolism disorders, and reduce oxidative stress; (3) 8-week aerobic exercise reduces skeletal muscle NOX4 expression and increases glucose tolerance; reduces the expression of LDH, PDH, SDH, and CCO in mouse skeletal muscle; increases the expression level of mitochondrial fusion protein 2 and PGC-1*α*; improves glucose tolerance; reduces energy metabolism of mouse skeletal muscle; reduces oxidative stress; and reduces insulin resistance. It is speculated that aerobic exercise can accelerate energy metabolism. This process may involve two aspects: firstly, increase the expression level of oxidative metabolism enzymes and promote the tricarboxylic acid cycle; secondly, increase the expression of Mfn2 and accelerate mitochondria fission or fusion to regulate energy metabolism, thereby reducing oxidative stress and insulin resistance.

## 1. Introduction

Impaired glucose tolerance (IGT) is an intermediate metabolic state between normal and diabetes [1], which accompanied with metabolic syndromes such as hyperinsulinemia, lipid metabolism disorders, and obesity [2]. IGT shows insulin resistant (IR) and will develop into type 2 diabetes mellitus (T2DM) [3]. People with IGT have moderate to severe muscle insulin resistance [4]; therefore, skeletal muscle reduces the uptake and utilization of glucose under the action of insulin.

Skeletal muscle is a main site of energy consumption and metabolism and plays a vital role in the body's metabolic balance. More and more reviews focused on that mitochondrial morphology, and bioenergetics may be linked to the etiology of IR in skeletal muscle [5–8]. Mitochondria is a dynamic reticulum of networked tubules that undergo fission and fusion, continuously [9, 10]. Mitochondrial dynamic behavior plays a vital role in bioenergetics function, cell viability, mitochondrial health, and quality control, and disruption of mitochondrial dynamics has been found in IR [8, 9, 11]. Mitochondrial dysfunction can lead to increase reactive oxygen species (ROS) synthesis, thereby affecting the basic function and structure of mitochondria, such as the mitochondrial respiratory chain, mitochondrial DNA, and mitochondrial membranes, as well as matrix proteins and related enzyme activities [11]. When the mitochondrial respiratory chain is affected, it will cause a vicious cycle, resulting in a decrease in the activity of the respiratory chain complex, synthesizing more ROS, and affecting ATP synthesis.

Insulin resistant mice on a long-term high-fat diet (HFD) showed a decline in skeletal muscle mitochondrial function [4], and it is mainly manifested as the expression of mitochondrial fission genes increased, while the expression of mitochondrial fusion genes decreased [12]. Mitochondrial fusion is a main factor for maintaining respiratory activity and then increase the bioenergetics capacity of the cells. Mitofusin 2 (Mfn2) identified to mediate mammalian mitochondrial fusion is a dynamin-related protein with GTPase activity located in the outer membrane of mitochondria, and it is abundantly expressed in muscle [13]. It is not only closely related to the fission and fusion of mitochondria but also plays an important role in mitochondrial morphology changing and mitochondrial function regulating [14, 15]. Mfn2 can stimulate substrate oxidation, respiration, and the expression of subunits involved in respiratory complexes [16]. Mfn2 repression can decrease the pyruvate and oxidation rates of glucose and reduces mitochondrial membrane potential in myotubes [17]. Subjected to an excess nutrient environment such as in type 2 diabetes or obesity increases mitochondrial fission and decreases mitochondrial fusion, which is closely related to uncoupled respiration [17]. Under normal circumstances, the expression of Mfn2 in muscle tissue is regulated by peroxisome proliferator-activated receptor *γ* coactivator-1*β* (PGC-1*β*), but when external stimuli and energy demand increase, peroxisome proliferator-activated receptor *γ* coactivator-1*α* (PGC-1*α*) participates in its regulation [18]. PGC is the main mechanism responsible for regulating the transcription of Mfn2.

Mitochondrial dysfunction is closely related to the occurrence and development of metabolic diseases. Exercise is an effective nonpharmaceutical intervention that can be used to manage and treat a wide range of lifestyle-related metabolic diseases [19, 20]. Aerobic exercise can improve skeletal muscle function and ameliorate IR, so aerobic exercise has important research value in the prevention and treatment of IGT. However, the mechanism how aerobic exercise ameliorates IR is still unclear. In the present study, we examine the skeletal muscle metabolic enzymes and the expression of protein NOX4, PGC-1*α*, and Mfn2 to provide potential mechanisms for the therapeutic effect of exercise on IGT treatment.

## 2. Materials and Methods

### 2.1. Animals

Two-week old male C57BL/6J mice used in this study were purchased from Wushi Experimental Animal Supply (Fuzhou, China). The mice were placed under standard controlled conditions (temperatures: 24–25°C, humidity: 45–55%, light: 12 h dark-night cycle), and after one week of acclimatization, animals were randomly divided into six different experimental groups (8 animals/group): (1) normal group (NOR), (2) normal control group (NC), (3) normal + exercise group (NE), (4) IGT group (IGT), (5) IGT control group (IC), and (6) IGT+ exercise group (IE). Mice in NOR were maintained on a regular diet (RD) (3.42 kcal/g, 22.47% kcal protein, 65.42% kcal carbohydrates, and 12.11% kcal fat) for 30 weeks. Mice in IGT were fed with a high-fat diet (HFD) (4.73 kcal/g, 20% kcal protein, 35% kcal carbohydrates, 45% kcal fat) for 30 weeks. Mice in the NC and NE groups received a regular diet, and mice in the IC and IE groups received a high-fat feeding for 30 weeks. After 30 weeks of rearing, mice in the NE and IE group receive 8 weeks of treadmill exercise. During these 8 weeks, the feeding conditions of mice in four groups were unchanged. Body weight was recorded every two weeks regularly. Intraperitoneal injection of glucose tolerance test (IPGTT) was conducted at the end of the 38th week. All experimental protocols conducted on the mice were approved by the Institutional Animal Care and Use Committee, Fujian Normal University (approval No.: IACUC-20190025). After the material collection, the experimental mice were euthanized by cervical dislocation method.

### 2.2. Treadmill

The mice in the exercise group received 12 m/min (75% maximum oxygen uptake) treadmill exercise intervention for 8 weeks at 60 min/time for 5 days/week, always in the afternoon. The speed of 8 m/min was as the initial speed and increased every min until the speed is 12 m/min, then performed at 12 m/min exercise intensity for 50 minutes; finally, the speed decreased every min until the speed is 8 m/min.

### 2.3. Detection of Serum Samples

For IPGTT, mice were fasted for 12 h and performed with an intraperitoneal injection of glucose load (2 g/kg of body weight), and glycemia was measured before injection and at 15, 30, 60, 90, and 120 min after injection. The area under the curve (AUC) was calculated for blood glucose (bg) during the IPGTT using the following equation: AUC = AUC = 0.5∗(bg0 + bg30)/2 + 0.5∗(bg30 + bg60)/2 + 1∗(bg60 + bg120)/2 [21]. The mice were fasted overnight and sacrificed at the 38th week. The fast blood glucose level was measured by glucometer (Jiancheng, Nanjing, China). The levels of lipid index (TG, TC, LDL-C, HDL-C), insulin, malondialdehyde (MDA), and superoxide dismutase (SOD) in serum were collectively tested using ELISA kits (Jiancheng, Nanjing, China), respectively, according to the manufactures' protocol. The homeostasis model assessment of insulin resistance (HOMA-IR) was calculated by fasting blood glucose (mmol/L) × fasting insulin (mIU/L)/22.5.

### 2.4. Western Blot Analysis

Total protein was extracted from the gastrocnemius in a RIPA-protease inhibitor PMSF cocktail (Beyotime) on ice and determined by the BCA method (P0009, Beyotime). An equal amount of protein samples (20 *μ*g) was separated by 10% sodium dodecyl sulfate–polyacrylamide gel electrophoresis (SDS-PAGE) and electrophoretically transferred to a polyvinylidene fluoride membrane (PVDF), and then, nonspecific binding sites were blocked with 5% (M/V) dissolved skimmed milk powder (Bio-Rad) at room temperature for 2 hours. Subsequently, the membranes probed, respectively, with anti-Mfn2 (86 kDa, 1 : 1000 dilution, Bioss, Beijing, China), anti-PGC-1*α* (91 kDa, 1 : 1000 dilution, Bioss, Beijing, China), anti-NOX4 (66 kDa, 1 : 1000 dilution, Bioss, Beijing, China), and anti-GAPDH (36 kDa, 1 : 1000 dilution, Bioss, Beijing, China) antibodies at 4°C overnight. Afterward, the incubation of horseradish peroxidase- (HRP-) conjugated secondary antibody (1 : 10000) was performed for 1 hour at 37°C. Finally, immunoreactive bands were detected by the ChemiImager 5500 V2.03 software. GAPDH was used to normalize the protein loading as an internal control.

### 2.5. Enzyme-Linked Immunosorbent Assay

Lactate dehydrogenase (LDH), pyruvate dehydrogenase (PDH), succinate dehydrogenase (SDH), and cytochrome *c* oxidase (CCO) of muscle tissue homogenate were detected by ELISA kit (SenBeiJia, Nanjing, China), respectively.

### 2.6. Statistical Analysis

Results were presented as means ± SE. Curve fitting was performed using the SigmaPlot 11.0 software (SYSTAT Software Inc., Chicago, IL, USA). Statistical significance was assessed using unpaired or paired Student's *t*-tests and ANOVA wherever appropriate. Differences were considered statistically significant by *p* < 0.05.

## 3. Results

### 3.1. High-Fat Diet Induced Impaired Glucose Tolerance in C57BL6J Mice

**Figure 1:**
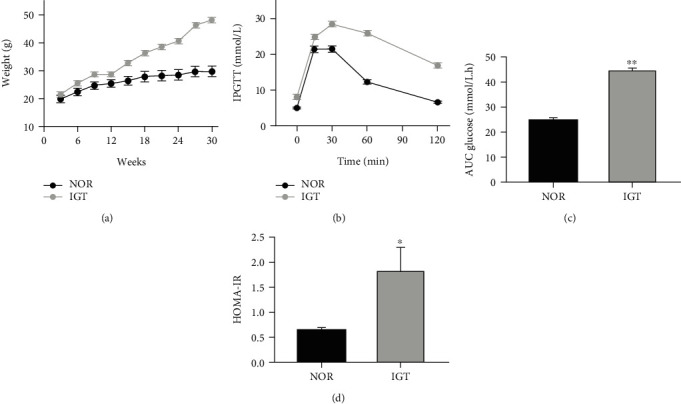
High-fat diet-induced impaired glucose tolerance in C57BL6J mice. (a) Body weight of two groups during fed with different diet. (b) Blood glucose response to an intraperitoneal glucose load after 30 weeks fed with normal diet and high-fat diet. (c) Area under the curve of blood glucose at the 30th week. (d) The HOMA-IR in two groups. Values are expressed as mean ± SE. *n* = 6. Compared with the control group (NOR), ^∗^*p* < 0.05 and ^∗∗^*p* < 0.01.

**Figure 2:**
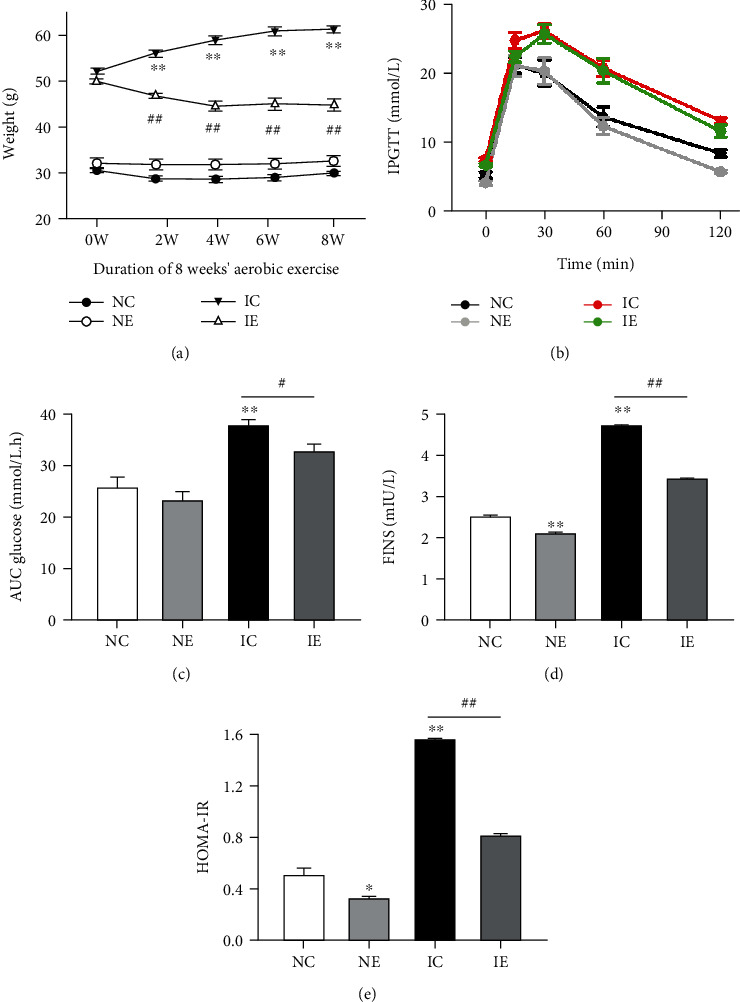
Aerobic exercise alleviates the impaired glucose tolerance (IGT) induced by high-fat diet. (a) Body weight of four groups (NC, NE, IC, IE). (b) Blood glucose response to an intraperitoneal glucose load after 8 weeks' aerobic exercise. (c) Area under the curve of blood glucose 8 weeks' aerobic exercise. (d, e) The fasting insulin and HOMA-IR in four groups. Values are expressed as mean ± SE. *n* = 6. Compared with the NC group, ^∗^*p* < 0.05 and ^∗∗^*p* < 0.01. Compared with the IC group, ^#^*p* < 0.05 and ^##^*p* < 0.01.

HFD was provided to induce IGT in C57BL6J mice, and the body weight was measured every 2 weeks. HFD mice show a significant increase in the body weight compared to the control (Figure 1(a)). After fed with HFD for 30 weeks, IPGTT was performed to identify the occurrence of glucose intolerance in HFD mice. As displayed in Figures 1(b) and 1(c), 2 h blood glucose level and the area under the curve (AUC) were enhanced significantly in HFD mice (IGT) compared to control mice (NOR) with regular diet (NOR: 24.87 ± 0.90, *n* = 3; IGT: 44.52 ± 1.14, *n* = 5, *p* < 0.05). Further analysis of HOMA-IR shows that HFD induces insulin resistance (NOR: 0.64 ± 0.05, *n* = 16; IGT: 1.83 ± 0.48, *n* = 45, *p* < 0.01) (Figure 1(d)). These suggested that HFD mimic the IGT model in vivo successfully.

### 3.2. Effects of High-Fat Diet on Lipid Metabolism in C57BL/6J Mice

To determine the effects of HFD on lipid metabolism in IGT mice, the levels of lipid index were employed in current study. The serum TC, TG, and LDH levels were increased, and the level of HDL was decreased (Table 1), which revealed that high-fat diet significantly increased lipogenesis in C57BL/6J mice.

### 3.3. Aerobic Exercise Alleviates the Impaired Glucose Tolerance (IGT) Induced by High-Fat Diet

The body weight was measured during 8 weeks of aerobic exercise. Moreover, 8 weeks of exercise training after 30 weeks of HFD feeding decreased body weight in the IE group compared with the IC group (Figure 2(a)). The level of 2 h blood glucose and AUC elevated in IC mice compared to NC mice (NC: 25.55 ± 2.11, *n* = 5; IC: 37.51 ± 1.33, *n* = 13, *p* < 0.01), and this situation was reversed after 8 weeks' aerobic exercise (IC: 37.51 ± 1.33, *n* = 13; IE: 32.51 ± 1.66, *n* = 9, *p* < 0.05) (Figures 2(b) and 2(c)). The insulin resistance in IE mice was assessed with an insulin tolerance test after 8 weeks' aerobic exercise, and fasting insulin (FINS) levels decreased in IE mice (IC: 4.69 ± 0.06, *n* = 4; IE: 3.40 ± 0.02, *n* = 4, *p* < 0.01) (Figure 2(d)), and the HOMA-IR values were lowered than in IC mice (IC: 1.74 ± 0.04, *n* = 4; IE: 0.91 ± 0.02, *n* = 4, *p* < 0.01) (Figure 2(e)). These results suggested that aerobic exercise could suppress IR and mitigate obesity in IC mice.

### 3.4. Aerobic Exercise Improves Lipid Metabolism

Aerobic exercise improved the lipid metabolism in IGT mice by reducing the levels of TC, TG, and low density lipoprotein (LDL-C) and increased the level of high density lipoprotein (HDL-C) (Table 2). Taken together, these results suggested that aerobic exercise promoted lipid metabolism, attenuated lipid accumulation of IGT mice, and stabilized lipid metabolism.

### 3.5. Aerobic Exercise Reduces Oxidative Stress Induced by High-Fat Diet

**Figure 3:**
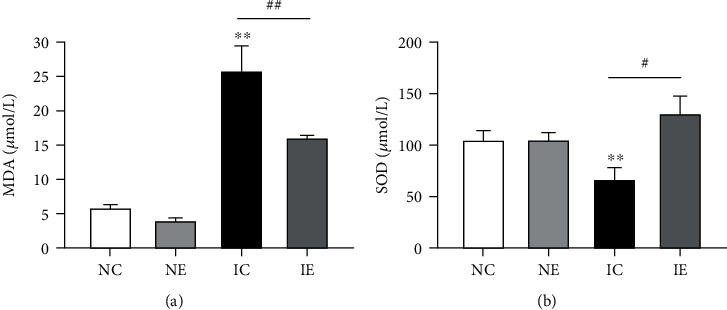
The MDA and SOD of serum detected in four groups. Data presented as mean ± SE. ^∗^*p* < 0.05 and ^∗∗^*p* < 0.01 compared with the NC group; ^#^*p* < 0.05 and ^##^*p* < 0.01 compared with the IC group.

MDA concentration is a biomarker of lipid peroxidation, and the study demonstrated that oxidative stress leads to lipid peroxidation leading by the formation of harmful products by MDA [22]. SOD is a main antioxidant enzyme responsible for ROS removal [23–25]. Thus, these two can be used as indicators to measure oxidative stress. The level of MDA was elevated in the IC group compared to NC significantly (NC: 5.72 ± 0.33 *μ*mol/L, *n* = 4; IC: 25.74 ± 1.93 *μ*mol/L, *n* = 4, *p* < 0.01), and aerobic exercise was significantly reduced the levels of MDA compared with the IC group (IC: 25.74 ± 1.93 *μ*mol/L, *n* = 4; IE: 15.80 ± 0.41 *μ*mol/L, *n* = 4, *p* < 0.01), while the level of SOD was lower in the IC group compared to NC significantly (NC: 103.59 ± 5.34 *μ*mol/L, *n* = 5; IC: 65.16 ± 8.19 *μ*mol/L, *n* = 5, *p* < 0.01), and aerobic exercise was significantly increased the level of SOD compared with the IC group (IC: 65.16 ± 8.19 *μ*mol/L, *n* = 3; IE: 129.82 ± 6.87 *μ*mol/L, *n* = 8, *p* < 0.01) (Figure 3).

### 3.6. Aerobic Exercise Increases Mitochondrial Oxidase Activity

**Figure 4:**
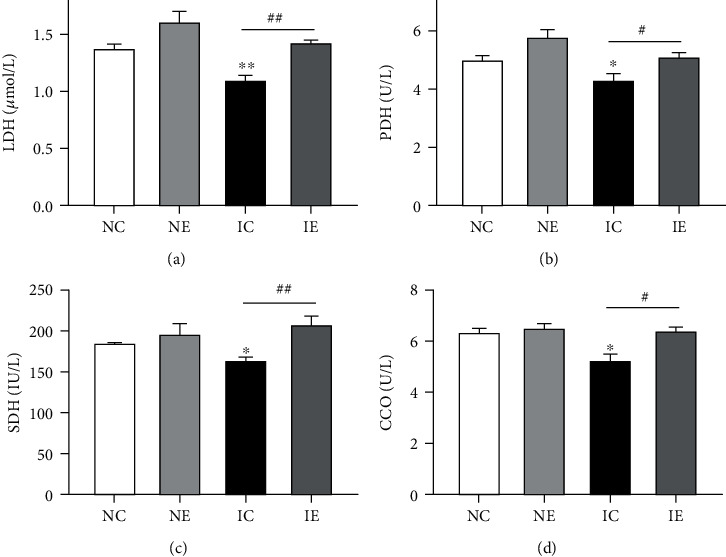
The concentration of LDH, PDH, SDH, and CCO detected in gastrocnemius tissue homogenate from four groups. Data presented as mean ± SE. ^∗^*p* < 0.05 and ^∗∗^*p* < 0.01 compared with the NC group; ^#^*p* < 0.05 and ^##^*p* < 0.01 compared with the IC group.

IR conditions are characterized by changes in mitochondrial activity in skeletal muscle which caused by reduction of mitochondrial mass or mitochondrial dysfunction, and mitochondria is involved in different kinds of biological processes in eukaryotic cell. One of the most important functions of mitochondria is oxidation ability. The activity of skeletal muscle mitochondrial oxidase (LDH, PDH, SDH, CCO) all decreased in the IC group compared to the NC group, while reversed after 8 weeks' aerobic exercise (Figure 4, and see Table 3 for specific data).

### 3.7. Aerobic Exercise Reverses the Expression of NOX4, PGC-1*α*, and Mfn2 in Skeletal Muscle of IGT Mice

**Figure 5:**
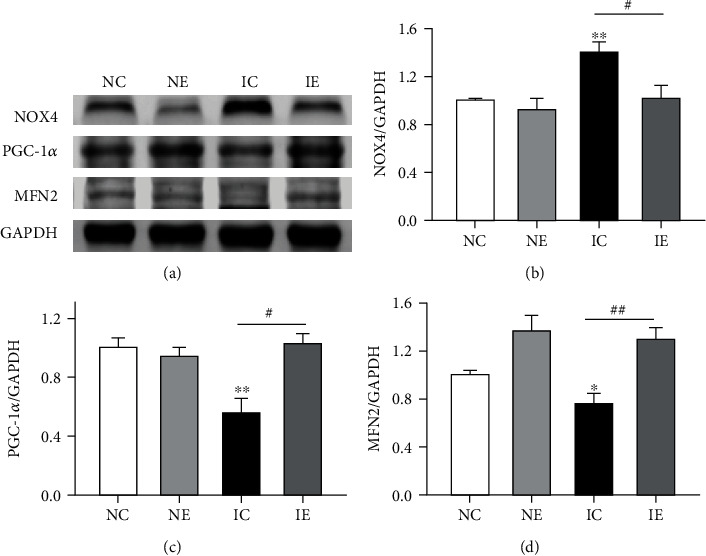
Expression of NOX4, PGC-1*α*, and Mfn2 in skeletal muscle with or without aerobic exercise. (a) Representative protein bands of NOX4, PGC-1*α*, and Mfn2. (b–d) Protein levels of NOX4, PGC-1*α*, and Mfn2 in the NC, NE, IC, and IE groups evaluated by Western blotting, with GAPDH as an internal control for normalization. Data presented as mean ± SE. ^∗^*p* < 0.05 and ^∗∗^*p* < 0.01 versus NC; ^**#**^*p* < 0.05 and ^##^*p* < 0.01 versus IC. Each group consisted of 4-6 rats.

The expressions of NOX4, PGC-1*α*, and Mfn2 were altered in skeletal muscle of IGT mice; NOX4 was increased in the IC group compared with the NC group, but PGC-1*α* and Mfn2 were decreased in the IC group. After 8-week aerobic exercise, these proteins in the IE group all reversed (Figure 5).

## 4. Discussion

Impaired glucose tolerance (IGT) is a progressive metabolic disease but not at the level defining diabetes, which is characterized by a higher glucose response to an intraperitoneal injection glucose than normal plasma [26, 27]. In our study, high-fat diet (HFD) was used to establish a model for IGT in C57BL/6J mice according to Surwit et al. [28], and then, we use 8-week aerobic exercise intervention to explore the effect on IGT mice. Our major findings are the following: (1) aerobic exercise alleviated the IGT in HFD induced mice, and the IPGTT experiment showed that the glucose intolerance was significantly alleviated after aerobic exercise according to the blood glucose level before and 2 h after the intraperitoneal injection of glucose; (2) aerobic exercise stabilized lipid metabolism and reduced oxidative stress; (3) 8-week aerobic exercise improves mitochondrial oxidation function of skeletal muscle in mice fed with high-fat diet; and (4) the expressions of NOX4, PGC-1*α*, and Mfn2 were altered in gastrocnemius muscle of IGT mice, and the changes of these proteins were reversed after 8-week aerobic exercise. Hence, our results, we emphasized the effects of aerobic exercise on skeletal muscle mitochondrial dynamics in HFD mice, and aerobic exercise is an effective treatment for alleviation of IGT.

In modern society, obesity due to HFD and physical inactivity can lead to various chronic diseases such as cardiovascular disease, type 2 diabetes, and cancer, all of which are associated with insulin resistance [29]. Skeletal muscle, with its mass and high rate of insulin-stimulated glucose transport, represents an essential metabolic tissue in the development of IR [30]. Thus, obesity-induced skeletal muscle dysfunction can lead to metabolic disorders, especially insulin resistance [31]. In addition, obesity can lead to abnormalities in skeletal muscle, including protein turnaround, glucose taker act reduction, lipid metabolic disorders, and mitochondrial dysfunction, which resulted in or from insulin resistance [32, 33]. As one of the important mechanisms for inducing insulin resistance, skeletal muscle mitochondria play a key role in regulating insulin resistance [34]. Merz et al. demonstrated that skeletal muscle-specific STX4 remediates HFD-induced insulin resistance via suppressing mitochondrial fission [35]. The reduced mitochondrial capacity in skeletal muscle is suggested to underlie IR development in obesity and type 2 diabetes [36]. Therefore, understanding the link between mitochondria and IR is important for developing treatments to alleviate IR-related skeletal muscle disease, thereby improving overall health.

Exercise as an effective nonpharmaceutical intervention can be used to manage and treat a wide range of lifestyle-related metabolic diseases [19, 20]. Regular aerobic and resistance exercise have been demonstrated to improve the metabolic disorders of diabetes and its complications by inducing body composition, glycemic control, insulin sensitivity, and controlling lipid profile [37–39]. In this study, HFD-induced mice displayed higher insulin resistance and lipid metabolism disorder, while 8-week aerobic exercise showed a compensatory effect on the alterations, so aerobic exercise alleviated the impaired glucose tolerance in high-fat diet-induced mice.

Malondialdehyde (MDA) concentration is a biomarker of lipid peroxidation, which is the result of oxidative stress [22]. As shown in Figure 5, aerobic exercise decreased the elevated MDA concentration in IGT mice, and the change of SOD which as an antioxidant is just the opposite. SOD as a member of antioxidant system converts the generated superoxide anion radical into hydrogen peroxide; then, reducing superoxide anions interacts with nitric oxide to form nitrite, so the main role of SOD is ROS scavengers in the body [23–25]. In addition, Leelarungrayub et al. reported that moderate-intensity aerobic dance for six weeks could reduce malondialdehyde (MDA) and increase total antioxidant capacity (TAC) among inactive women [40]. Mice fed with high-fat diet after 8 weeks of swimming training, the expression of SOD in skeletal muscle significantly increased, and the expression of MDA significantly decreased [41]. These results confirm that aerobic exercise improves the body's antioxidant capacity. Studies also have shown that aerobic exercise is an important potential strategy to increase the oxidation capacity of skeletal muscle mitochondria [42]. This is consistent with our results that aerobic exercise increases the activity of mitochondrial oxidase in skeletal muscle (Figure 3).

Mitochondria are important energy metabolism organelles located in the double-layer membrane structure of eukaryotic cells, which are places for cell aerobic respiration and oxidative phosphorylation. The function of mitochondrial aerobic respiration is completed by the respiratory chain on the inner mitochondrial membrane, and mitochondrial respiratory chain is the main place for mitochondria to synthesize ATP and also for ROS generation. In our study, aerobic exercise improves the function of mitochondrial respiratory chain in skeletal muscle from HFD mice (Figure 4). In contractile skeletal muscle, the production of ROS mainly originates from NOX4 [43, 44]. Studies have pointed out that inhibitors of NOX4 can increase insulin sensitivity [45], and swimming exercise can reduce the NOX4 level in an IR model [46]. Our results also show that aerobic exercise reduces the expression of NOX4 in the muscles from IGT mice, and these all indicate that 8 weeks of aerobic exercise can reduce the level of oxidative stress in IGT mice.

PGC-1*α* is an important transcriptional coactivator that regulates cell energy metabolism. PGC-1*α* expression and its cotranscription activity were reduced in skeletal muscle of humans with T2DM or prediabetic individuals [47]. Transgenic mice overexpressing PGC-1*α* in muscle show an increased mitochondrial mass [48]. After a long period of exercise (high-speed oxidation type especially), the expression of PGC-1*α* can be increased in skeletal muscle [49, 50]. Our results shown that the expression of PGC-1*α* decreased in muscle from HFD-induced IGT mice and reversed after aerobic exercise (Figure 5(b)). PGC-1*α* is not only the main regulator of mitochondrial biogenesis [51, 52] but also other regulators acting on mitochondrial quality control systems (fusion/fission and mitophagy) [53]. PGC-1*α* controls the expression of Mfn2 through interaction with the transcription factor ERR*α* [18, 54]. Mfn2 is highly expressed in skeletal and is crucial for mitochondrial fusion [14]. The function of mitochondria depends on their quality control, and an essential of this quality control is the high plasticity of their dynamic structure, which enables them to change continuously by fusion and fission processes [9]. The balance between mitochondrial fusion and fission can be broken through lipid accumulation, resulting in mitochondrial dysfunction such as loss of mitochondrial membrane potential, reduction of oxygen consumption, and elevation of ROS production. Studies have pointed out that when a null mutation occurs in the Mfn2 gene, the expression of Mfn2 decreases, and the mitochondrial fusion in the cell is inhibited, resulting in cell dysfunction which include the reduction of glucose oxidation level, the conversion of cell energy metabolism to glycolysis, the tricarboxylic acid cycle, and the electron transport chain restrained [55]. When the expression of Mfn2 increases, it can increase cellular oxidative phosphorylation and glycogen utilization [17]. Mfn2 expression in skeletal muscle is decreased in high-fat diet-induced obese mice; thereby, mitochondrial respiratory function and ATP content are decreased [56]. Studies have shown that high-fat diet induces IR in rats, which is related to the decrease of Mfn2 expression in skeletal muscle tissue, and the production of ROS increased [57]. Mfn2 deficiency causes mitochondrial dysfunction, which leads to enhance ROS production. This study found that the expression of Mfn2 protein in skeletal muscle from IGT mice was lower than that from the NC group, and the concentration of skeletal muscle mitochondrial oxidases LDH, PDH, SDH, and CCO was also significantly reduced. After 8 weeks of aerobic exercise intervention, IGT mice skeletal muscle Mfn2 protein expression was significantly increased, and the concentration of mitochondrial oxidases LDH, PDH, SDH, and CCO was reversed, indicating that 8-week aerobic exercise can reduce the oxidative stress level of IGT mice and improve mitochondrial function by increasing the expression of Mfn2 in skeletal muscle. It was well known that exercise training can enhance muscle mitochondrial function and improve systemic metabolic homeostasis [58]. Exercise activates the signal network to control mitochondrial remodeling coordinately, including mitochondrial biogenesis, mitophagy, and dynamics [59].

To sum up, upregulation of mitochondrial mass and function, also referred to as mitochondrial biogenesis, is instrumental in exercise training-induced improvement of skeletal muscle function and whole body metabolic homeostasis [60, 61]. Skeletal muscle, with its mass and high rate of insulin-stimulated glucose transport, represents an essential metabolic tissue in the development of IR [30]. Exercise increases mitochondrial biogenesis and mitochondrial oxidase content in skeletal muscle, reduces oxidative stress, and improves muscle function. Thus, exercise improves high-fat diet-induced systemic insulin resistance partly by enhancing skeletal muscle metabolic capacity. We believe that aerobic exercise is a suitable method to stimulate skeletal muscle cells, to cause adaptive changes in cell structure and function, to stabilize the dynamic balance of mitochondrial fusion and fission, and to promote mitochondrial function, thereby reduce insulin resistance.

## 5. Conclusion

Several studies have reported that exercise maintains the balance between mitochondrial fusion and fission under normal conditions, but few studies have assessed the impact of exercise on IGT-induced dysfunction of mitochondrial dynamics in skeletal muscles. Our study provides a possible mechanism: aerobic exercise increases the expression level of metabolic enzymes, accelerates the metabolism of energy by promoting the tricarboxylic acid cycle, reduces the generation of oxidative stress, increases the Mfn2 protein, promotes mitochondrial fusion, and improves energy metabolism, thereby improving IGT oxidative stress and insulin resistance.

## Figures and Tables

**Table 1:** Glucolipid metabolism in the NOR and IGT groups (M ± SE, *n* = 3-4).

Indexes	NOR	IGT
TC (mmol/L)	4.10 ± 0.45	11.14 ± 0.87^∗∗^
TG (mmol/L)	0.52 ± 0.07	0.98 ± 0.05^∗∗^
LDL-C (mmol/L)	0.31 ± 0.02	1.67 ± 0.16^∗∗^
HDL-C (mmol/L)	1.65 ± 0.10	0.96 ± 0.17^∗^

Data presented as mean ± SE. Compared with the NOR group, ^∗^*p* < 0.05 and ^∗∗^*p* < 0.01.

**Table 2:** Glucolipid metabolism in IGT mice after 8 weeks (M ± SE, *n* = 3-8).

Indexes	NC	NE	IC	IE
TC (mmol/L)	3.13 ± 0.32	2.70 ± 0.55	13.14 ± 0.63^∗∗^	6.88 ± 0.20^##^
TG (mmol/L)	0.35 ± 0.05	0.28 ± 0.03	0.63 ± 0.06^∗^	0.40 ± 0.05^#^
LDL-C (mmol/L)	0.36 ± 0.02	0.26 ± 0.02	2.14 ± 0.39^∗∗^	1.05 ± 0.08^#^
HDL-C (mmol/L)	0.35 ± 0.05	0.28 ± 0.03	0.63 ± 0.06^∗^	0.40 ± 0.05^#^

Data presented as mean ± SE. ^∗∗^*p* < 0.01 compared with the NC group; ^#^*p* < 0.05 and ^##^*p* < 0.01 compared with the IC group.

**Table 3:** The changes of skeletal muscle mitochondrial oxidase in IGT mice after 8 weeks (M ± SE, *n* = 3-8).

Indexes	NC	NE	IC	IE
LDH (*μ*mol/L)	1.38 ± 0.04	1.61 ± 0.11	1.09 ± 0.05^∗∗^	1.41 ± 0.04^##^
PDH (U/L)	4.98 ± 0.18	5.73 ± 0.30	4.26 ± 0.27^∗^	5.06 ± 0.20^#^
SDH (IU/L)	182.13 ± 4.31	195.9 ± 13.59	162.5 ± 5.44^∗^	205.83 ± 12.44^##^
CCO (U/L)	6.25 ± 0.25	6.45 ± 0.26	5.13 ± 0.32^∗^	6.33 ± 0.19^#^

Data presented as mean ± SE. ^∗^*p* < 0.05 and ^∗∗^*p* < 0.01 compared with the NC group; ^#^*p* < 0.05 and ^##^*p* < 0.01 compared with the IC group.

## Data Availability

The animal experiment data used to support the findings of this study are available from the corresponding author upon request.
